# Tumor-suppressive miR-218-5p inhibits cancer cell proliferation and migration via EGFR in non-small cell lung cancer

**DOI:** 10.18632/oncotarget.8576

**Published:** 2016-04-04

**Authors:** Kegan Zhu, Hanying Ding, Wengong Wang, Zhicong Liao, Zheng Fu, Yeting Hong, Yong Zhou, Chen-Yu Zhang, Xi Chen

**Affiliations:** ^1^ State Key Laboratory of Pharmaceutical Biotechnology, Collaborative Innovation Center of Chemistry for Life Sciences, NJU Advanced Institute for Life Sciences (NAILS), Jiangsu Engineering Research Center for MicroRNA Biology and Biotechnology, School of Life Sciences, Nanjing University, Nanjing, Jiangsu 210046, China; ^2^ Department of Thoracic and Cardiovascular Surgery, Department of General Surgery, Affiliated Drum Tower Hospital of Nanjing University Medical School, Nanjing, Jiangsu 210008, China

**Keywords:** NSCLC, miR-218-5p, EGFR, proliferation, migration

## Abstract

Lung cancer remains the leading cause of cancer-related death worldwide, and non-small cell lung cancer (NSCLC) accounts for approximately 80% of lung cancer cases. Recently, microRNAs (miRNAs) have been consistently demonstrated to be involved in NSCLC and to act as either tumor oncogenes or tumor suppressors. In this study, we identified a specific binding site for miR-218-5p in the 3′-untranslated region of the epidermal growth factor receptor (EGFR). We further experimentally validated miR-218-5p as a direct regulator of EGFR. We also identified an inverse correlation between miR-218-5p and EGFR protein levels in NSCLC tissue samples. Moreover, we demonstrated that miR-218-5p plays a critical role in suppressing the proliferation and migration of lung cancer cells probably by binding to EGFR. Finally, we examined the function of miR-218-5p *in vivo* and revealed that miR-218-5p exerts an anti-tumor effect by negatively regulating EGFR in a xenograft mouse model. Taken together, the results of this study highlight an important role for miR-218-5p in the regulation of EGFR in NSCLC and may open new avenues for future lung cancer therapies.

## INTRODUCTION

Lung cancer remains the leading cause of cancer-related death in the world, and the 5-year survival rates and recurrence rates differ greatly depending on the pathologic stage [1]. Lung cancer includes two major histological types, i.e., small cell lung cancer (SCLC) and non-small cell lung cancer (NSCLC). NSCLC accounts for approximately 80% of all lung cancer subtypes [2]. NSCLC has led the way in the development of targeted therapies because various biomarkers have been discovered and applied to clinic treatments, and the epidermal growth factor receptor (EGFR) is the most dominant of these biomarkers [3, 4]. The accumulation of EGFR in NSCLC has been widely reported, and EGFR signaling is essential for the initiation and progression of NSCLC [1, 2, 5]. During the last decade, different EGFR tyrosine kinase inhibitors (TKIs) have been developed, and three main inhibitors (gefitinib, erlotinib and afatinib) have been effectively approved for individuals with NSCLC [5]. Currently, EGFR-TKI treatments are the standard first-line therapies for patients with advanced NSCLCs harboring activating EGFR mutations [6]. However, these therapeutic agents are ultimately limited by the emergence of secondary EGFR mutations and other molecular mechanisms that confer drug resistance [7, 8]. Novel targeted drugs that efficiently inhibit all of these mutations of EGFR in NSCLC patients are urgently needed.

miRNAs are a class of 19-24-nucleotide-long non-coding RNAs that repress gene expression in two manners: by inhibiting mRNA translation or promoting mRNA degradation. During the last few years, increasing evidence has indicated that miRNAs are involved in a wide range of biological processes, including cell proliferation, apoptosis and migration [9–11]. Regarding cancers, miRNAs have also been found to play important roles as either tumor suppressors or oncogenes according to their expression levels and the involved downstream targets [12–14]. Recently, miR-218-5p was reported to act as a tumor suppressor in many human cancers, such as bladder cancer, hepatocellular carcinoma, gastric cancer, oral cancer and renal cell carcinoma [15–19]. In NSCLC, miR-218-5p is also a potential tumor suppressor [20, 21], but the precise molecular mechanism through which miR-218-5p influences NSCLC progression remains largely unknown, which indicates that further investigations are required.

In this study, we identified EGFR as a target gene of miR-218-5p. We also detected an inverse correlation between miR-218-5p and EGFR protein levels in NSCLC tissues. The direct inhibition of EGFR translation by miR-218-5p and the potential role of miR-218-5p as a tumor suppressor in NSCLC development have been experimentally validated *in vitro* and *in vivo*.

## RESULTS

### EGFR is inhibited by miR-218-5p in the NSCLC cell lines A549 and H1975

**Figure 1:**
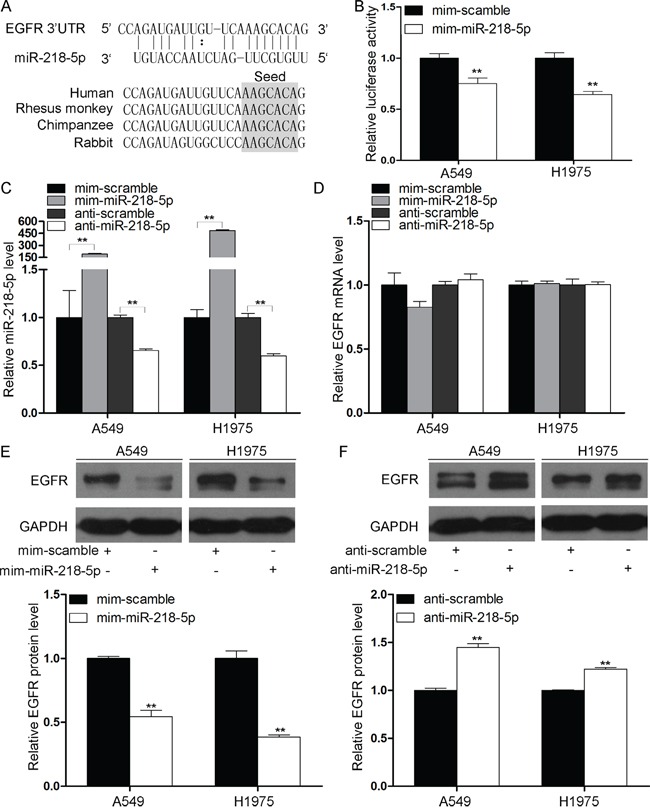
miR-218-5p directly downregulates EGFR in NSCLC cells **A.** Schematic representation of the putative miR-218-5p binding sequence on the 3′-UTR of the EGFR mRNA. **B.** The luciferase reporter plasmid encoding the full-length 3′-UTR of EGFR was co-transfected into the A549 and H1975 cells along with miR-218-5p mimic or scrambled mimic. The luciferase activities were normalized to the β-galactosidase levels of the control (**P < 0.01). **C.** Quantitative RT-PCR analyses of the expression levels of miR-218-5p in the A549 and H1975 cells transfected with miR-218-5p mimic, scrambled mimic, antisense miR-218-5p or scrambled antisense miRNA. The results were normalized to U6 (**P < 0.01). **D.** Quantitative RT-PCR analyses of the expression levels of EGFR mRNA in the A549 and H1975 cells after transfection. The results were normalized to GAPDH. **E** and **F.** Western blotting analyses of the expression levels of EGFR protein in the A549 and H1975 cells after transfection. Upper panel: representative images; lower panel: quantitative analysis (**P < 0.01).

Previous studies have demonstrated that miR-218-5p functions as a tumor suppressor in many cancers [15–19], but its targets in NSCLC are largely unknown. Here, we used a bioinformatics method (http://www.targetscan.org) to predict the target genes that miR-218-5p may bind to. From all of the candidates, we selected EGFR as a potential target because EGFR plays an important role in NSCLC development. As shown in Figure 1A, a predicted hybridization was identified between miR-218-5p and the 3′-UTR of EGFR. Furthermore, the miR-218-5p binding sequence in the EGFR 3′-UTR was highly conserved across species (Figure 1A). To confirm whether miR-218-5p could bind to EGFR, a luciferase reporter assay was performed. The 3′-untranslated region (3′-UTR) of EGFR was fused downstream of the firefly luciferase gene in a reporter plasmid, and the recombination plasmid was transfected into NSCLC cell lines A549 and H1975 along with an miR-218-5p mimic. The miR-218-5p mimic was transfected into cells to induce the overexpression of miR-218-5p, and a scrambled mimic was simultaneously transfected into cells as a negative control. As expected, the luciferase activity was remarkably reduced in the cells that were co-transfected with the luciferase reporter plasmid and the miR-218-5p mimic (Figure 1B).

Furthermore, we performed quantitative RT-PCR and western blotting to investigate the effects of miR-218-5p overexpression and knockdown on EGFR mRNA and protein levels. An antisense miR-218-5p was transfected into the cells to knock down miR-218-5p, and a scrambled antisense miRNA was simultaneously transfected as a negative control. First, we measured the miR-218-5p levels in the A549 and H1975 cells following transfection with the miR-218-5p mimic or antisense miR-218-5p. As expected, the miR-218-5p expression levels were significantly increased in the A549 and H1975 cells that were transfected with the miR-218-5p mimic and dramatically reduced in the cells that were transfected with the antisense miR-218-5p (Figure 1C). Consequently, although the expression levels of EGFR mRNA were unaffected by the overexpression or knockdown of miR-218-5p (Figure 1D), the expression levels of the EGFR protein were significantly inhibited by the introduction of miR-218-5p in the A549 and H1975 cells (Figure 1E), and the antisense miR-218-5p significantly increased the EGFR protein levels in the A549 and H1975 cells (Figure 1F). Taken together, these results demonstrate that miR-218-5p negatively regulated the expression of EGFR by directly binding to the 3′-UTR of EGFR and inhibiting EGFR translation in NSCLC cell lines.

### miR-218-5p is downregulated and EGFR is upregulated in NSCLC tissues

**Figure 2:**
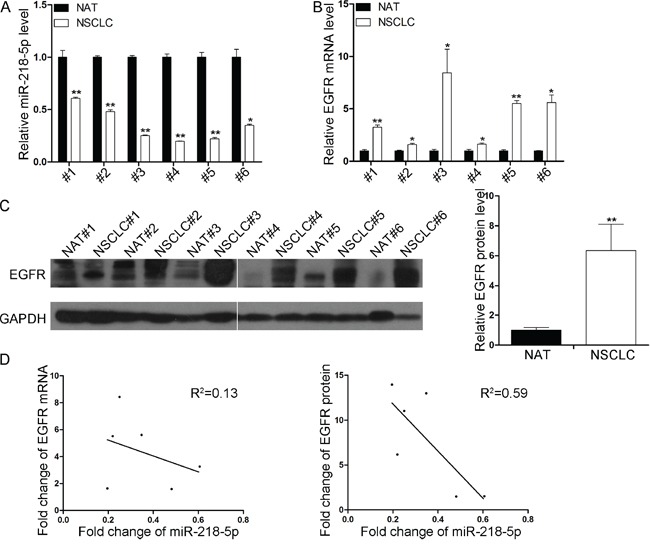
Inverse correlation between the miR-218-5p and EGFR protein expression levels in NSCLC tissues **A.** Quantitative RT-PCR analyses of the expression levels of miR-218-5p in 6 pairs of NSCLC and NAT samples. The results were normalized to U6 (*P < 0.05, **P < 0.01). **B.** Quantitative RT-PCR analyses of the expression levels of EGFR mRNA in 6 pairs of NSCLC and NAT samples. The results were normalized to GAPDH (*P < 0.05, **P <0.01). **C.** Western blotting analyses of the expression levels of EGFR protein in 6 pairs of NSCLC and NAT samples. Left panel: representative image; right panel: quantitative analysis (**P < 0.01). **D.** Pearson's correlation scatter plot of the fold change in miR-218-5p and EGFR mRNA (left) or protein (right) in human NSCLC tissues.

Because miRNAs are generally thought to exhibit expression patterns that are opposite to those of their targets, we next investigated the expression patterns of miR-218-5p and EGFR in NSCLC tissues and normal adjacent tissues (NATs). The expression levels of miR-218-5p were consistently lower in the NSCLC tissues compared with the NATs from the same patient (Figure 2A). In contrast, the EGFR mRNA and protein levels were consistently higher in the cancer tissues (Figure 2B and 2C). Next, the inverse correlations of the miR-218-5p levels with the EGFR protein and mRNA levels were further analyzed using Pearson's correlation scatter plots. The results revealed that the inverse correlation of miR-218-5p with the EGFR protein was stronger than that with the EGFR mRNA in the NSCLC tissues (Figure 2D). The inverse correlation between the miR-218-5p and EGFR protein levels in the NSCLC tissues further indicated that EGFR is a direct target of miR-218-5p.

### miR-218-5p suppresses the migration and proliferation of NSCLC cells

**Figure 3:**
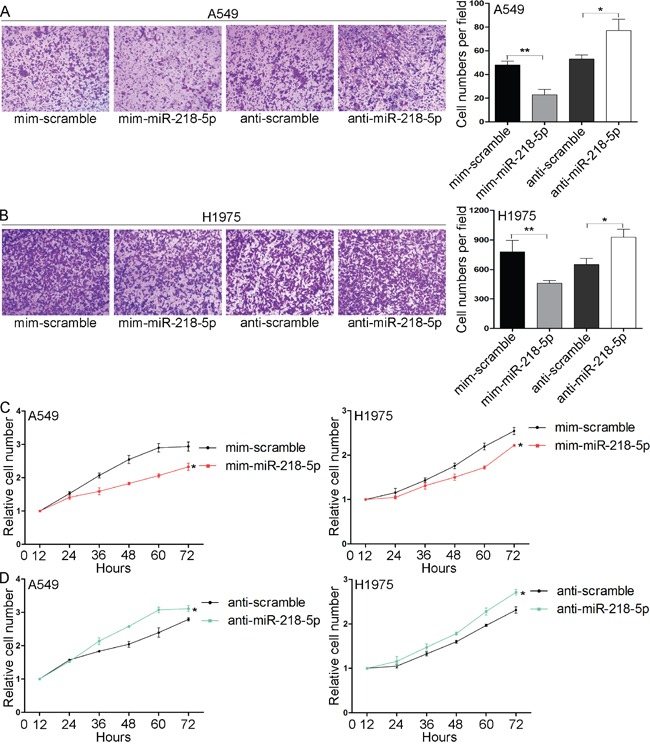
miR-218-5p inhibits the migration and proliferation of NSCLC cells *in vitro* **A** and **B.** Transwell analyses of the migrated A549 (A) and H1975 (B) cells transfected with the miR-218-5p mimic, scrambled mimic, antisense miR-218-5p or scrambled antisense miRNA. A549 cells were allowed to migrate for 6 h and H1975 cells were allowed to migrate for 12 h. Left panel: representative images showing cells that migrated through the filter; right panel: cell numbers counted in each field (*P < 0.05; **P < 0.01). **C** and **D.** CCK8 analyses of the proliferation rates of the A549 (C) and H1975 (D) cells transfected with the miR-218-5p mimic, scrambled mimic, antisense miR-218-5p or scrambled antisense miRNA (*P <0.05).

To further explore whether miR-218-5p affected the tumorigenesis of NSCLC cells, we performed migration (transwell) and cell proliferation assays (Cell Counting Kit 8, CCK8) in A549 and H1975 cells. The transwell assay revealed that the migratory capabilities were greatly decreased in the A549 and H1975 cells that were transfected with the miR-218-5p mimic and increased in the A549 and H1975 cells transfected with the antisense miR-218-5p (Figure 3A and 3B). Furthermore, The CCK8 assays revealed that the cell proliferation abilities were significantly inhibited following the overexpression of miR-218-5p in the A549 and H1975 cells (Figure 3C), whereas the knockdown of miR-218-5p in the A549 and H1975 cells promoted the cell growth rate (Figure 3D). Overall, these results suggest that miR-218-5p exerts anti-proliferative and anti-migratory effects on NSCLC cells and therefore may act as a tumor suppressor in NSCLC.

**Figure 4:**
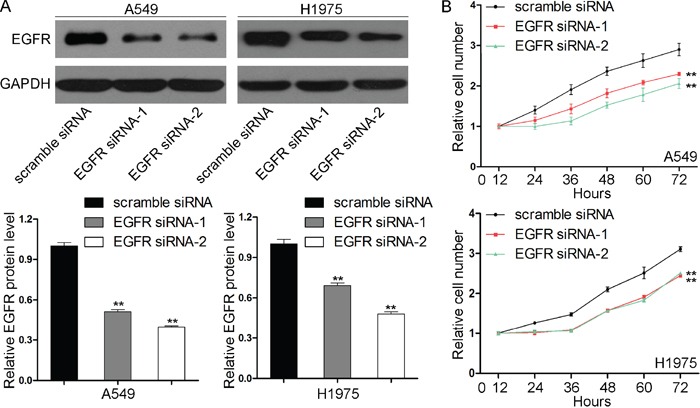
Effects of EGFR knockdown on the proliferation of NSCLC cells **A.** Western blotting analyses of the expression levels of the EGFR protein in the A549 and H1975 cells transfected with scrambled siRNA or EGFR siRNAs (EGFR siRNA-1 and EGFR siRNA-2). Upper panel: representative images; lower panel: quantitative analysis (**P < 0.01). **B.** CCK8 analyses of the proliferation rates of the A549 (upper) and H1975 (lower) cells transfected with scrambled siRNA or EGFR siRNAs (**P < 0.01).

We next analyzed the biological consequences of EGFR inhibition in NSCLC cells. Western blotting and quantitative RT-PCR assays confirmed that the EGFR protein levels were efficiently reduced when siRNA-mediated gene silencing was performed in the A549 and H1975 cells (Figure 4A). In support of the notion that EGFR is essential to the promotion of tumorigenesis, the transfection of EGFR siRNA markedly reduced the proliferation abilities of A549 and H1975 cells (Figure 4B), which suggests that the inhibition of cell proliferation by EGFR knockdown was similar to that elicited by miR-218-5p overexpression. Thus, miR-218-5p may suppress the proliferation and migration of NSCLC cells by binding to EGFR.

### miR-218-5p inhibits the growth of NSCLC xenografts *in vivo* by regulating EGFR

**Figure 5:**
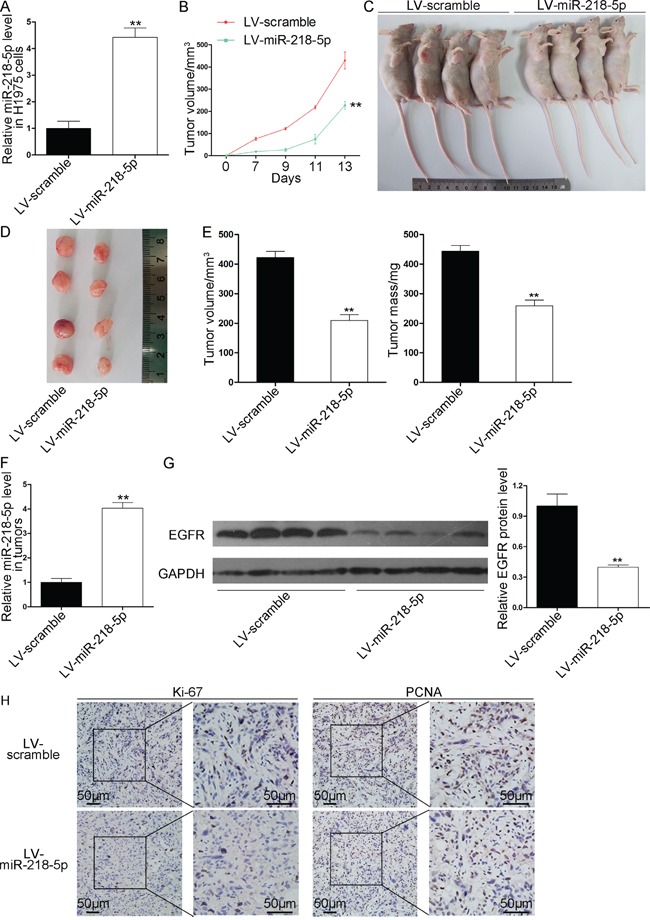
Effects of miR-218-5p on the growth of NSCLC xenografts in mice H1975 cells were infected with a control lentivirus or a lentivirus causing the overexpression of miR-218-5p. The infected H1975 cells (5 × 10^6^ cells per mouse) were implanted subcutaneously into nude mice (10 mice per group). Beginning on day 7 after implantation, the tumor lengths and widths were measured every 2 days for a total of 4 measurements, and the tumor growth curves were evaluated. At 13days after implantation, the tumors were dissected, and the exact sizes and weights were measured. **A.** Quantitative RT-PCR analyses of the miR-218-5p levels in the H1975 cells after infection with a control lentivirus or a lentivirus causing the overexpression of miR-218-5p (**P < 0.01). **B.** Tumor growth curves in the implanted mice (**P < 0.01). **C** and **D.** Representative images of the nude mice bearing xenograft tumors (C) and the excised tumors (D). **E.** Quantitative analyses of the tumor volumes and weights (**P < 0.01). **F.** Quantitative RT-PCR analyses of the miR-218-5p levels in the tumors from the implanted mice (**P < 0.01). **G.** Western blotting analyses of the EGFR protein levels in the tumors from the implanted mice. Left panel: representative images; right panel: quantitative analysis (**P < 0.01). **H.** Immunohistochemical staining analyses of Ki-67 and PCNA in the tumors from the implanted mice.

Finally, we evaluated the effects of miR-218-5p on the growth of NSCLC xenografts in nude mice. H1975 cells were infected with either a lentiviral expression vector to overexpress miR-218-5p or a negative control lentiviral vector. Efficient overexpression of miR-218-5p in the H1975 cells following lentiviral infection was verified by quantitative RT-PCR (Figure 5A). Next, the infected H1975 cells were subcutaneously implanted into nude mice. Beginning on day 7 after implantation, the tumor lengths and widths were measured every 2 days for 4 measurements. The tumor growth curve revealed a significant retardation in the miR-218-5p-overexpressing group compared with the control group (Figure 5B). Subsequently, the tumors were dissected, and the exact sizes and weights were evaluated. Compared with the control group, the mean volume and mass of the tumors in the miR-218-5p-overexpressing group were significantly smaller and lighter (Figure 5C-5E). Subsequently, the total RNA and protein were extracted from each tumor and used to evaluate the expression levels of miR-218-5p and EGFR. After 13 days of xenograft growth *in vivo*, the tumors from the miR-218-5p-overexpressing group exhibited significant increase in miR-218-5p expression compared with the tumors from the control group (Figure 5F). Consequently, the EGFR protein levels were dramatically reduced in the miR-218-5p-overexpressing group (Figure 5G). Additionally, the proliferative activities of the tumor cells were assessed via immunohistochemical staining for Ki-67 and PCNA. The Ki-67 and PCNA staining intensities were decreased in the tumors from the miR-218-5p-overexpressing group (Figure 5H). In summary, these results suggest that miR-218-5p possesses tumor-suppressing activity and may repress NSCLC development by negatively regulating EGFR expression.

## DISCUSSION

Recently, an important role of miRNAs in the genesis and progression of human cancers has emerged. Many researchers have reported the extensive alteration of miRNA expression in the initial and developmental stages of human cancers, and miRNAs are involved in tumor suppression and promotion [12–14]. The importance of miRNA function and dysfunction in various human cancers suggests that modulation of miRNA expression may serve as a novel therapeutic modality for such diseases. To date, there are three main approaches that have been used in potential miRNA-targeting therapy: expression vectors (miRNA sponges), small-molecule inhibitors and antisense oligonucleotides. Chemically synthesized miRNAs or oligonucleotides that target miRNAs have already been proved to efficiently inhibit cancer development [22, 23]. Furthermore, several preclinical and clinical trials of miRNA-targeting therapies are in the process and may pave the way for cancer therapy [24, 25].

In NSCLC, there are several miRNAs that are essential for tumor development, including miR-9, let-7 and miR-193a-3p [26–28]. In this study, we demonstrated that miR-218-5p was downregulated in NSCLC tissues and could suppress cell proliferation and migration *in vitro* and retard tumor growth *in vivo*, which suggests a role for miR-218-5p as a tumor suppressor gene in NSCLC. In agreement with this hypothesis, miR-218-5p has been found to be significantly downregulated and behave as a tumor-suppressing miRNA in numerous human cancer types, including bladder cancer, hepatocellular carcinoma, gastric cancer, oral cancer and renal cell carcinoma [15–19]. To explore the molecular mechanism through which miR-218-5p contributes to cancer suppression, we provided evidence that the targeting of EGFR may be the pathway by which miR-218-5p exerts its tumor-suppressing function. Therefore, the modulation of EGFR by miR-218-5p may explain why the downregulation of miR-218-5p during NSCLC carcinogenesis can promote cancer progression.

With the substantial advances in our understanding of tumor biology, key signaling pathways that are involved in mediating NSCLC growth and progression have been identified. Dominant oncogenes involved in the pathogenesis of NSCLC have attracted substantial interest, and their central roles and fundamental contributions to the misbehavior of NSCLC cells have become clear. One such gene is EGFR, whose activation is thought to be important in the development of NSCLC [29]. NSCLC is associated with EGFR overexpression in up to 80% of patients, and high EGFR gene copy numbers are found in nearly 60% of the cases [30, 31]. The EGFR signaling pathway is importantly implicated in tumor cell growth, migration, angiogenesis, metabolism and metastasis [32–36]. In addition to NSCLC, the amplification of EGFR expression and signaling is a common feature in a variety of human cancers including prostate, gastric, ovarian, colorectal, renal, breast, glioma and head and neck cancers [37–39]. In recent years, with the help of the development of some drugs that specifically target EGFR, the landscape of NSCLC therapy has been significantly altered. There are two major types of anti-EGFR therapeutics: humanized antibodies directed against the extracellular domain of EGFR and small-molecule TKIs that compete with ATP in the tyrosine-kinase domain of EGFR [40]. However, many patients who initially respond to EGFR-targeted treatment eventually develop drug resistance that may be due to secondary EGFR mutations or other resistance mechanisms [41, 42]. Hence, future studies should focus on the development of new drugs to overcome and prevent resistance [43]. Considering that miR-218-5p is an upstream regulator of EGFR, it is possible to upregulate miR-218-5p to contain EGFR *in vivo*. The re-expression of miRNAs that are lost in cancers is a novel therapeutic strategy for cancers, and efforts to predictably alter oncogene profiles by increasing specific miRNAs through either transfection or viral delivery methods have demonstrated the potential utilities of miRNAs as therapeutic molecules in the treatment of human cancers. Future research emphasis is needed to characterize the feasibility of targeting miR-218-5p in NSCLC therapy and developing simplified and cost-effective manipulation methods.

Taken together, our results reveal a critical role for miR-218-5p as a tumor suppressor in NSCLC carcinogenesis which functions through the repression of EGFR translation. This study may provide a potential novel therapeutic strategy for NSCLC in the future.

## MATERIALS AND METHODS

### Cell lines and clinical specimens

The human lung cancer cell lines A549 and H1975 were purchased from the Shanghai Institute of Cell Biology, Chinese Academy of Sciences (Shanghai, China). The A549 cells were maintained in F-12K medium (Invitrogen, Carlsbad, CA, USA) supplemented with 10% fetal bovine serum (FBS, GIBCO, CA, USA), and the H1975 cells were maintained in RPMI 1640 medium supplemented with 10% FBS. All cells were cultured in a 37°C water-saturated atmosphere with 5% CO_2_. The NSCLC tumors and paired normal adjacent tissues were derived from patients undergoing surgical procedures at the Affiliated Drum Tower Hospital of Nanjing University Medical School (Nanjing, China). All of the patients provided written consent, and the ethics committee of Nanjing University approved all aspects of this study. The specimens were immediately frozen in liquid nitrogen at the time of surgery and stored at -80°C. The patient information and clinical features are listed in Supplementary Table 1.

### RNA extraction and quantitative RT-PCR

The total RNA of the cultured cells and human tissues were extracted using TRIzol reagent (Invitrogen) according to the manufacturer's instructions. Assays for the quantification of the miRNAs were performed using TaqMan miRNA probes (Applied Biosystems, Foster City, CA) according to the manufacturer's instructions. Briefly, 1 μg of total RNA was reverse-transcribed to cDNA using AMV reverse transcriptase (TaKaRa, Dalian, China) and a stem-loop RT primer (Applied Biosystems). Real-time PCR was performed using a TaqMan PCR kit on an Applied Biosystems 7500 Sequence Detection System (Applied Biosystems). All of the reactions were run in triplicate. The reaction conditions and analysis methods were performed as described previously [44]. The relative levels of miRNAs in the cells and tissues were normalized to U6. To quantify the EGFR mRNA, 1 μg of total RNA was reverse-transcribed to cDNA using EGFR reverse primer and AMV reverse transcriptase (TaKaRa) in the reaction, which was performed with the following conditions: 16°C for 15 min, 42°C for 60 min, and 85°C for 5 min. Next, real-time PCR was performed with the RT product, SYBER Green Dye (Invitrogen) and specific primers for EGFR and GAPDH. The sequences of the primers were as follows: EGFR forward: 5′-TTGCCGCAAAGTGTGTAACG-3′; EGFR reverse: 5′-GTCACCCCTAAATGCCACCG-3′; GAPDH forward: 5′-CGAGCCACATCGCTCAGACA-3′; and GAPDH reverse: 5′-GTGGTGAAGACGCCAGTGGA-3′. The reactions were incubated at 95°C for 5 min followed by 40 cycles of 95°C for 15 s and 60°C for 1 min. After the reactions, the C_T_ values were determined by setting a fixed threshold. The relative amount of EGFR mRNA was normalized to GAPDH.

### Overexpression and knockdown of miR-218-5p

The synthetic miR-218-5p mimic, scrambled mimic, antisense miR-218-5p, scrambled antisense miRNA and EGFR siRNAs were purchased from Genepharma (Shanghai, China). The cells were seeded in 6-well plates on the first day. When the cells had grown to approximately 70% confluent, the transfections were performed with Lipofectamine 2000 (Invitrogen) following the manufacturer's instructions. For each well, 100 pmol mimic, antisense or siRNA was transfected. The cells were harvested 48 h after transfection for quantitative RT-PCR and western blotting. The siRNA sequences were as follows: EGFR siRNA-1: AACACAGUGGAGCGAAUUCCU; and EGFR siRNA-2: CGCAAAGUGUGUAACGGAAUA.

### Luciferase reporter assay

For the luciferase reporter assay, the entire 3′-UTR of the human EGFR transcript variant 4 was amplified and inserted into the pMIR-reporter plasmid (Ambion, Austin, TX, USA). The cells were cultured in 12-well plates, and each well was transfected with 0.8 μg of firefly luciferase reporter plasmid, 0.8 μg of a β-galactosidase (β-gal) expression plasmid (Ambion), and equal amounts (40 pmol) of miR-218-5p mimic or scrambled mimic using Lipofectamine 2000 (Invitrogen). The β-gal plasmid was used as a transfection control. Twenty-four hours post-transfection, the cells were assayed using a luciferase assay kit (Promega, Madison, WI, USA).

### Western blotting

The transfected cells were rinsed with PBS (pH 7.4) and lysed in RIPA Lysis buffer (Beyotime, China) supplemented with Protease and Phosphatase Inhibitor Cocktail (Thermo Scientific 78440) on ice for 30 min. The tissue samples were frozen solid with liquid nitrogen, ground into a powder and lysed in RIPA Lysis buffer containing the Protease and Phosphatase Inhibitor Cocktail. The cell lysates or tissue homogenates were centrifuged for 10 min (12000 g, 4°C). The supernatant was collected, and the protein concentration was calculated with a Pierce BCA protein assay kit (Thermo Scientific, Rockford, IL, USA). Equivalent quantities of protein were separated on 10% SDS–PAGE gels and transferred to a PVDF membrane (Millipore, Bedford, MA, USA). After blocking with 5% nonfat milk at room temperature for 1 h, the membranes were immunostained with primary antibodies at 4°C overnight, washed three times in TBST, and then incubated with secondary antibody at room temperature for 1 h. Band signals were detected with an enhanced chemiluminescence reagent (Cell Signaling Technology Inc., USA). The following primary antibodies were used: EGFR (R&D systems, USA, 1:1000) and GAPDH (Santa Cruz Biotechnology, Santa Cruz, CA, USA, 1:2000).

### Cell viability assay

To assess cell viability, the transfected cells were seeded into 96-well plates at a density of 5000 cells per well. The cell proliferation indices were measured using CCK8 assays (Dojindo, Japan) at 12, 24, 36, 48, 60 and 72 h after the cells were seeded. The absorbance was determined at 450 nm.

### Cell migration assay

For the transwell assays, 24-well plates with 8.0-μm pore size polycarbonate membranes (BD Biosciences, USA) were used. A total of 5×10^4^ cells were suspended in 100 μl of serum-free medium and seeded on the upper chamber. Then, 600 μl medium containing 10% FBS was added to the bottom chamber. The whole chambers were incubated at 37°C for 6 h (for the A549 cells) or 12 h (for the H1975 cells). Then, the cells in the top chamber were removed with cotton swabs, and the cells on the lower membrane surface were fixed with 4% paraformaldehyde and stained with 0.1% crystal violet. Finally, the cells were counted under a microscope at 100× magnification.

### *In vivo* tumor xenograft studies

Six-week-old male nude mice were purchased from the Model Animal Research Center of Nanjing University (Nanjing, China) and maintained under specific pathogen-free conditions at Nanjing University. H1975 cells were infected with either the miR-218-5p overexpressing lentivirus or the control lentivirus. After 48 h, the cells were injected subcutaneously into the nude mice (5×10^6^ cells per mouse, 10 mice per group). Beginning on day 7, the lengths and widths of the tumors were measured every 2 days for a total of 4 measurements. Then, the mice were dissected, and the tumors were separated. Simultaneously, the lengths, widths and weights of the tumors were accurately measured. The volumes were calculated as follows: volume = 1/4 × (length) × (width)^2^. Subsequently, total RNA and protein were extracted from the tumor for quantitative RT-PCR and western blotting. Tumor section slides were subjected to immunohistochemical analysis using Ki-67 and PCNA staining according to the manufacturer's instructions. All animal care and handling procedures were performed in accordance with the National Institutes of Health's Guide for the Care and Use of Laboratory Animals and were approved by the Institutional Review Board of Nanjing University (Nanjing, China).

### Statistical analysis

All experiments were independently repeated at least three times. The quantitative RT-PCRs, luciferase reporter assays and CCK8 assays were performed in triplicate. All data are presented as the means ± the SEs. The statistical analyses were performed using IBM SPSS Statistics 19 and GraphPad Prism 5. The differences were considered statistically significant at P < 0.05 based on Student's t-tests.

## SUPPLEMENTARY TABLE


