# Supplementary material for: Tumor-suppressive miR-218-5p inhibits cancer cell proliferation and migration via EGFR in non-small cell lung cancer
Source: Oncotarget. 2016 Apr 4;7(19):28075–85. doi: 10.18632/oncotarget.8576 (PMC5053710; doi:10.18632/oncotarget.8576)
Supplement: Supplementary file 1 [file oncotarget-07-28075-s001.pdf]

SUPPLEMENTARY TABLE

Supplementary Table S1: Clinical features of lung cancer patients

	Age	Gender	Tumor subtype	Pathological Stage
Case #1	57	Male	Squamous cell carcinoma	IB
Case #2	56	Male	Adenocarcinoma	
Case #3	64	Male	Squamous cell carcinoma	IB
Case #4	59	Male	Squamous cell carcinoma	
Case #5	64	Female	Adenocarcinoma	IA
Case #6	60	Male	Squamous cell carcinoma	IIB
